# Catering of high-risk foods and potential of stored food menu data for timely outbreak investigations in healthcare facilities, Italy and Germany

**DOI:** 10.1017/S0950268823000468

**Published:** 2023-03-22

**Authors:** Idesbald Boone, Michele Luca D'Errico, Luigi Iannetti, Gaia Scavia, Rosangela Tozzoli, Steen Ethelberg, Tim Eckmanns, Klaus Stark, Hendrik Wilking, Sebastian Haller

**Affiliations:** 1 Department of Infectious Disease Epidemiology, Robert Koch Institute, Berlin, Germany; 2 Department of Food Safety, Nutrition and Veterinary Public Health, Istituto Superiore di Sanità, Rome, Italy; 3 Istituto Zooprofilattico Sperimentale dell'Abruzzo e del Molise “G. Caporale”, National Reference Laboratory for *Listeria monocytogenes*, Teramo, Italy; 4 Infectious Disease Epidemiology and Prevention, Statens Serum Institut, Copenhagen, Denmark

**Keywords:** Electronic data, food-borne infections, healthcare-associated infections, health facilities, outbreaks

## Abstract

Healthcare-associated foodborne outbreaks (HA-FBOs) can cause significant morbidity and mortality, affecting particularly vulnerable hospital populations. Electronic records of food served in healthcare facilities (HCFs) could be useful for timely investigations of HA-FBOs. We explored the availability and usability of electronic food menu data to support investigations of HA-FBOs through a survey among 35 HCFs in Germany (*n* = 13) and in Italy (*n* = 22). Large variability was reported in the storage time of menu data (from no storage up to 10 years) and their formats, including paper, electronic (PDF, Word, Excel), or fully searchable databases (15/22 in Italian HCFs, 3/13 in German HCFs). Food products that may present a risk to vulnerable persons – including deli salads, raw/fermented sausage products, soft cheese, smoked fish or frozen berries – were offered on the menu of all HCFs in Germany, and one-third of the Italian HCFs. The usability of electronic food menu data for the prevention or investigation of HA-FBOs may be suboptimal in a large number of HCFs in Germany, as well as in some HCFs in Italy. Standardised collection for use of electronic food menu data might help discover the association between illnesses and food eaten during outbreak investigations. Hospital hygienists, food safety and public health authorities should collaborate to increase implementation of food safety guidelines.

## Introduction

In 2020, a total of 3086 foodborne outbreaks, including 20 017 cases and 37 deaths, were reported by the European Union member states to the European Food Safety Authority [1]. Among foodborne outbreaks, healthcare-associated foodborne outbreaks (HA-FBOs) are of public health concern. Indeed, a literature review on HA-FBOs in 37 member countries of the Organisation for Economic Cooperation and Development retrieved 85 outbreaks occurring between 2001 and 2018, which were mainly associated with the consumption of food contaminated with *Salmonella* (24 outbreaks), norovirus (22 outbreaks) and *Listeria* (19 outbreaks) [2]. HA-FBOs result from the consumption of contaminated food served in healthcare facilities (HCFs) and represent a risk, especially for vulnerable patients (children up to the age of 5 years, elderly people, pregnant, immunosuppressed) [2, 3].

HA-FBOs are likely underreported due to the lack of systematic surveillance of foodborne outbreaks in HCFs, since food is rarely considered a potential vehicle for healthcare-associated outbreaks, as compared to other routes of transmission [2]. Moreover, outbreaks with relatively low numbers of cases distributed across different HCFs or protracted outbreaks (e.g. listeriosis outbreaks) can only be detected by systematic surveillance and by routine whole-genome sequencing [4]. Examples of these include a listeriosis outbreak in Germany involving 13 cases associated with the consumption of meat in HCFs [4], a listeriosis HA-FBO in the UK with nine cases in different HCFs linked to the consumption of contaminated sandwiches provided by a common supplier [5] and a listeriosis HA-FBO in Italy among cancer and immunocompromised patients likely due to a contaminated meat slicer in the hospital kitchen [6]. Other HA-FBOs among vulnerable patients were reported and associated with food considered high-risk for vulnerable patients in HCFs, such as (raw) pork products [7], oysters [8] or (uncooked) frozen berries [9].

Usually, in outbreak investigations, food menu data are obtained through patient interviews, which are often resource-intensive. Furthermore, this is subject to inaccurate patient recall of previously consumed meals [10], especially in outbreaks caused by pathogens with a long incubation time like *Listeria monocytogenes* and hepatitis A virus, possibly leading to failures in investigations or implication of the contaminated food product. In community settings, consumption purchase data (e.g. credit card data on food purchases) have been successfully used as an alternative or complementary data source to support outbreak investigations [11]. Likewise, in an outbreak of listeriosis in different hospitals in Australia in 2013, an electronic menu database (which records all hospital food menu items ordered by patients during their admissions) allowed investigators to rapidly identify potential food sources [12].

In Italy, a national guideline for food catering in hospitals, HCFs and schools was published by the Ministry of Health in 2021, which addresses various aspects of food service, including food safety [13]. The need for food business operators to store food menu data or to keep reference samples of food is not explicitly mentioned in the guideline above. However, this is frequently requested from food business operators in the food service contracts by the institutions that manage and control the service, such as the hospitals and the local health units.

In Germany, the food safety sector provides food safety recommendations to minimise the risk of contaminated food in hospital kitchens [14] and food safety recommendations for communal facilities with a focus on vulnerable groups [3]. Furthermore, the public health and hospital infection control sectors publish hygiene requirements when handling food in HCFs [15] and for immunosuppressed patients [16]. Although there is no legal obligation for caterers in HCFs to store reference samples, except in the case of monitoring of zoonoses and zoonotic pathogens, there are recommendations in Germany to do so [17].

In this study, we investigated the data availability, accessibility and usability of food menu data in Italian and German HCFs; we wanted to identify possible gaps and provide recommendations to better identify food vehicles associated with HA-FBOs.

## Methods

### Study setting

We conducted a survey among HCFs, jointly in Italy and in Germany, between June and November 2019, as well as in February 2021. The survey was addressed to the direction of the HCFs and completed by hospital hygienists, kitchen managers, caterers or dieticians in charge of managing the food menus for the patients.

In Italy, a convenience sample consisted of 22 HCFs; 14 HCFs were selected by the Istituto Superiore di Sanità (ISS) and eight HCFs were selected by the Istituto Zooprofilattico Sperimentale dell'Abruzzo e del Molise (IZSAM). The first 14 HCFs consisted mainly of second-level paediatric hospitals (including special and reference clinical units) all over Italy. These hospitals were selected due to the vulnerability of patients to foodborne infections, and to investigate special aspects of hospital catering policies and food consumption in a specific patient population such as paediatrics. In addition, a few general hospitals were selected among the hospitals interested in establishing an active surveillance for hospital-acquired viral infections. The latter eight HCFs were selected by the IZSAM because of existing contacts and consisted of secondary hospitals and nursing homes in the Abruzzo and Molise regions.

In Germany, a convenience sample consisted of 13 HCFs; six HCFs that had previously participated in a project on healthcare-associated infections in long-term care facilities and seven HCFs (primary, tertiary and specialised care hospitals) that had been collaborating in projects and studies with the Robert Koch Institute.

### Data collection and questionnaire

In Italy, the same questionnaire was administered through different approaches: self-administration of a semi-structured online questionnaire using LimeSurvey for paediatric hospitals, and face-to-face interviews using a semi-structured static (paper) questionnaire administered with the participating HCFs in the Abruzzo and Molise regions. In Germany, the questionnaire was designed as an Adobe Acrobat form to facilitate self-administration by the participating HCFs.

The core questionnaire covered information on catering service management (in-house catering: all food preparation steps from raw ingredients to the final food served to patients undertaken within the facility; external catering: all the services, preparation and cooking steps undertaken by an external company; mixed catering: in-house catering combined with external service providers), format and storage duration of food menu data, availability of food menu data for each patient, history of food menu data by the HCF in relation to a suspected foodborne outbreak and information on whether the HCF provided known high-risk foods, such as deli salads, raw/fermented sausage products, soft cheese, smoked fish and frozen berries [3]. We did not include questions related to food preparation, storage conditions or overall hygiene practices, as we did not have indicators to objectify the answers.

Questions on reference food samples were added only in the questionnaire in Germany, as storage of reference food samples in HCFs is recommended in parts of Germany [17].

### Data analysis

Questionnaire data were only analysed descriptively (frequency and proportions).

## Results

In total, 35 HCFs (22 in Italy and 13 in Germany) participated in our survey, including 26 hospitals (19 in Italy and seven in Germany) and nine nursing homes (three in Italy and six in Germany) of various sizes, according to bed capacity (Table 1).

**Table 1.** Characteristics of participating HCFs in Italy and Germany and availability of food menu data

	Hospital				Nursing home			
	Italy (*n* = 19)	%	Germany (*n* = 7^a^)	%	Italy (*n* = 3)	%	Germany (*n* = 6)	%
Number of beds								
≤200	9	47	2	29	2	67	5	83
201–800	9	47	2	29	1	33	1	17
>800	1	5	3	43				
Catering system								
In-house^b^	1	5	3	43	1	33	5	83
External^c^	5	26	3	43	0	0	0	0
Mixed^d^	13	69	1	14	2	67	1	17
Link of food menu data with individual patients								
Link with all patients	17	89	3	43	1	33	1	17
Link only with specific patients	0	0	0	0	1	33	2	33
No link	2	11	3	43	1	33	3	50
N/A			1	14				
Format of menu data^e^								
No stored data	0	0	1	14	0	0	1	17
Paper	11	58	0	0	3	100	2	33
Electronic (PDF, Word, Excel)	7	37	3	43	0	0	2	33
Fully searchable database	15	79	3	43	0	0	0	0
N/A			1	14	0	0	1	17
Storage time of menu data								
<1 month	3	16	1	14	0	0	0	0
≥1 month and <1 year	4	21	1	14	2	67	1	17
≥1 year	10	53	2	29	1	33	3	50
No menu data storage/until discharge	1	5	0	0	0	0	1	17
N/A	1	5	3	43			1	17

N/A, no answer.

a Two questionnaires included information about a hospital combined with a nursing home served by the same caterer. Only the information related to the hospital was retained in the analysis.

b All food preparation steps from raw ingredients to the final food served to patients were prepared in-house by the HCF.

c Outsourcing: all the services, preparation and cooking steps were undertaken by an external company.

d In-house catering, combined with external service providers (e.g. external company that works in the hospital kitchen).

e Multiple answers possible.

Catering systems included in-house, external and mixed catering. Catering activities (mixed and external catering) were mainly outsourced by Italian hospitals (18/19 hospitals), whereas in Germany, in-house catering was more often reported in hospitals (3/7) and in nursing homes (5/6) compared to the Italian hospitals (1/19) and nursing homes (1/3).

The majority (17/19) of hospitals in Italy reported that a direct link between the food menu data and individual patients (i.e. documentation of patient-specific food menu choices) could be established, in contrast to half of the participating hospitals in Germany. In nursing homes, the direct link of food menu data to individual nursing home residents was uncommon both in Italy (1/3) and in Germany (1/6).

Heterogeneity also existed in food menu data formats, and ranged from paper, electronic (PDF, Word, Excel), to fully searchable electronic databases (e.g. as part of commercial software used for catering management). Electronic databases were available for most of the Italian hospitals (15/19), in contrast to the German hospitals (3/7). No electronic databases were used by the nursing homes of our study.

The storage duration of menu data differed considerably between HCFs, ranging from no storage up to 10 years. We asked the German HCFs whether they collected information on who ordered but did not eat an ordered meal. This question was only answered by nine HCFs (four hospitals and five nursing homes); of these nine, only three nursing homes collected this information.

**Figure 1.**
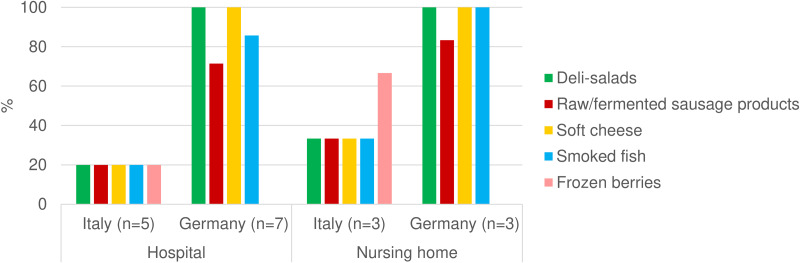
Proportion of HCFs offering potentially high-risk food on their menu in Germany (*n* = 10) and in Italy (*n* = 8). Responses from Italian HCFs originated from Abruzzo and Molise regions only.

High-risk foods were offered on the menu in 3/8 Italian HCFs from the Abruzzo and Molise regions, as well as in all German HCFs (Fig. 1). One hospital in Germany, in which a previous HA-FBO occurred due to the consumption of spreadable raw fermented sausage (German Teewurst [18]), did not offer this food on its menu anymore, whereas other potentially high-risk foods were still offered to patients; for example, soft cheese and smoked fish.

In Germany, reference food samples from the lunch meals were taken by 11/13 HCFs. The storage time reported for these reference samples ranged from less than 7 to more than 20 days.

## Discussion

### Food menu data in HCFs

The survey highlighted that the availability of patient-linked food menu data, data formats (paper, electronic data/PDF, searchable databases) and data storage duration were highly heterogeneous between the investigated HCFs and the two countries. In particular, it was not possible to link food menu items to individual patients in about half of the participating German HCFs and 14% of the Italian HCFs. We found that food menu data may not be currently analysed to support outbreak investigations in a large number of HCFs in Germany, as well as in some HCFs in Italy. A single HCF in Italy reported that food menu data were previously used in a suspected foodborne outbreak investigation; analysing these food menu data, the researchers concluded that the norovirus was likely transmitted human-to-human, and not foodborne [19]. One HA-FBO reported by a German hospital was associated with the consumption of spreadable raw fermented sausage (German Teewurst) contaminated with *Salmonella* Derby, affecting very old patients in hospitals or elderly care homes [18]. In this outbreak, food menu data were not available, as the spread was typically offered with other options, buffet style, with bread for breakfast and cold dinner.

To increase their usability for outbreak purposes, food menu data should be documented for all offered meals (e.g. breakfast, lunch and dinner) and be unequivocally linked to individual patients or nursing home residents. Despite the lack of a legal obligation to store food menu data, a minimum duration of storage of menu data (at least 1 year) would be crucial for the investigation of protracted outbreaks such as listeriosis outbreaks or for food with a long shelf-life, such as frozen food. For instance, in a listeriosis HA-FBO in Germany in 2019 [4], electronic food menu data were insufficiently available and not patient-specific to support the analysis of detailed food exposure data. Furthermore, specific information about ‘consumed meals’ instead of only ‘ordered meals’ would be beneficial. For data that are already collected in digital format, storage costs have considerably fallen in recent years. Further digitisation of hospital services, including IT solutions that allow faster and differentiated data on patient meal requests to the kitchen, may be expected [20]. The digitisation and collection of additional data will result in additional costs, including those for human resources. The cost–benefit of collecting and digitising food menu data in HCFs should be evaluated, since usage and analysis of these data may have shared benefits for different healthcare professionals such as dieticians, caterers and infectious diseases specialists, as well as for increasing patient satisfaction (subjective rating of hospital food services quality) [21].

Concerning the storage of food reference samples, it would make sense to collect samples of all meals (breakfast, lunch and dinner). However, for protracted outbreaks, the storage of reference samples may be impractical due to limited storage capacities.

### Safe food in HCFs

The current survey suggests that despite existing food safety recommendations [12], patients and nursing home residents are exposed to food considered to be of high risk for HA-FBO among vulnerable patients. Further research is needed to identify whether the presence of such food items on the menu is related to a lack of knowledge of food safety recommendations and/or reflects a demand by the patients and nursing home residents, as well as to assess whether these foods are also effectively offered to specific vulnerable patient groups (e.g. immunocompromised) in HCFs. Previous food monitoring among 1880 HCFs by the German Food Safety Authorities in 2017 also highlighted a lack of knowledge of recommendations about high-risk food by 45% of the participating HCFs [22].

In the current study, a nursing home in Germany indicated, as a reason for not participating in the survey, that ‘food poisoning is not an issue’ in their HCF, highlighting the need to both increase awareness about the risk of HA-FBOs and to strengthen food hygiene recommendations among staff and food business operators in HCFs.

### Limitations

The main limitation of our study is that a small convenience sample of German and Italian HCFs was used, and that the distribution of HCF types and sizes may not be representative. It should be noted that the answers to the questionnaire were self-reported by the respondents of the HCFs. A larger representative follow-up survey is needed to achieve more explanatory powers also regarding differences between and within nursing homes and hospitals of different sizes, organisational structures and healthcare levels.

To demonstrate the use of food menu data of HCFs for outbreak investigations, we would need to compare HCFs with linked patient–food menu data to HCFs without linked patient–food menu data in outbreak situations. As hospital-acquired infections are not that frequent, simulations may be the first step.

## Conclusions

We aimed to explore the availability, accessibility and usability of food menu data in HCFs to support the identification of food vehicles associated with HA-FBOs. We found that food menu data analyses to support outbreak investigations is challenging in Italy and in Germany due to incomplete documentation. As the survey suggests knowledge gaps on existing food safety recommendations in HCFs, we recommend further training to increase compliance with recommendations. In Italy, the results of this study were discussed with the healthcare professionals who participated in the survey in an online workshop, also as a measure to reduce knowledge gaps.

It may be worthwhile to explore whether electronic bedside meal ordering systems, which already have the potential to improve dietary intake and patient satisfaction [21], may as well provide good opportunities to store patient food menu data. Hopefully, the digitisation opportunities that occurred during the COVID-19 pandemic [23] will also be used to accelerate developments towards further digitisation of food menu data in HCFs. This will be a prerequisite to better assess the burden of contaminated food items in HA-FBOs.

## Data Availability

The dataset generated and/or analysed during the current study is available at the Zenodo repository, https://zenodo.org/record/5944854.
